# Independent Component Analysis in Spiking Neurons

**DOI:** 10.1371/journal.pcbi.1000757

**Published:** 2010-04-22

**Authors:** Cristina Savin, Prashant Joshi, Jochen Triesch

**Affiliations:** Frankfurt Institute for Advanced Studies, Frankfurt am Main, Germany; RIKEN Brain Science Institute, Japan

## Abstract

Although models based on independent component analysis (ICA) have been successful in explaining various properties of sensory coding in the cortex, it remains unclear how networks of spiking neurons using realistic plasticity rules can realize such computation. Here, we propose a biologically plausible mechanism for ICA-like learning with spiking neurons. Our model combines spike-timing dependent plasticity and synaptic scaling with an intrinsic plasticity rule that regulates neuronal excitability to maximize information transmission. We show that a stochastically spiking neuron learns one independent component for inputs encoded either as rates or using spike-spike correlations. Furthermore, different independent components can be recovered, when the activity of different neurons is decorrelated by adaptive lateral inhibition.

## Author Summary

How the brain learns to encode and represent sensory information has been a longstanding question in neuroscience. Computational theories predict that sensory neurons should reduce redundancies between their responses to a given stimulus set in order to maximize the amount of information they can encode. Specifically, a powerful set of learning algorithms called Independent Component Analysis (ICA) and related models, such as sparse coding, have emerged as a standard for learning efficient codes for sensory information. These algorithms have been able to successfully explain several aspects of sensory representations in the brain, such as the shape of receptive fields of neurons in primary visual cortex. Unfortunately, it remains unclear how networks of spiking neurons can implement this function and, even more difficult, how they can learn to do so using known forms of neuronal plasticity. This paper solves this problem by presenting a model of a network of spiking neurons that performs ICA-like learning in a biologically plausible fashion, by combining three different forms of neuronal plasticity. We demonstrate the model's effectiveness on several standard sensory learning problems. Our results highlight the importance of studying the interaction of different forms of neuronal plasticity for understanding learning processes in the brain.

## Introduction

Independent component analysis is a well-known signal processing technique for extracting statistically independent components from high-dimensional data. For the brain, ICA-like processing could play an essential role in building efficient representations of sensory data [1]–[4]. However, although many algorithms have been proposed for solving the ICA problem [5], only few consider spiking neurons. Moreover, the existing spike-based models [6], [7] do not answer the question how this type of learning can be realized in networks of spiking neurons using local, biologically plausible plasticity mechanisms (but see [8]).

Classic ICA algorithms often exploit the non-Gaussianity principle, which allows the ICA model to be estimated by maximizing some non-Gaussianity measure, such as kurtosis or negentropy [5]. A related representational principle is sparse coding, which has been used to explain various properties of V1 receptive fields [9]. Sparse coding states that only a small number of neurons are activated at the same time, or alternatively, that each individual unit is activated only rarely [10]. In the context of neural circuits, it offers a different interpretation of the goal of the ICA transform, from the perspective of metabolic efficiency. As spikes are energetically expensive, neurons have to operate under tight metabolic constraints [11], which affect the way information is encoded. Moreover, experimental evidence supports the idea that the activity of neurons in V1 is sparse. Close to exponential distributions of firing rates have been reported in various visual areas in response to natural scenes [12].

Interestingly, certain homeostatic mechanisms are thought to regulate the distribution of firing rates of a neuron [13]. These intrinsic plasticity (IP) mechanisms adjust ionic channel properties, inducing persistent changes in neuronal excitability [14]. They have been reported for a variety of systems, in brain slices and neuronal cultures [14], [15] and they are generally thought to play a role in maintaining system homeostasis. Moreover, IP has been found to occur in behaving animals, in response to learning (see [14] for review).

From a computational perspective, it is believed that IP may maximize information transmission of a neuron, under certain metabolic constraints [13]. Additionally, we have previously shown for a rate neuron model that, when interacting with Hebbian synaptic plasticity, IP allows the discovery of heavy-tailed directions in the input [16]. Here, we extend these results for a network of spiking neurons. Specifically, we combine spike-timing dependent plasticity (STDP) [17]–[19], synaptic scaling [20] and an IP rule similar to [16], which tries to make the distribution of instantaneous neuronal firing rates close to exponential.

We show that IP and synaptic scaling complement STDP learning, allowing single spiking neurons to learn useful representations of their inputs for several ICA problems. First, we show that output sparsification by IP together with synaptic learning is sufficient for demixing two zero mean supergaussian sources, a classic formulation of ICA. When using biologically plausible inputs and STDP, complex tasks, such as Foldiák's bars problem [21], and learning oriented receptive fields for natural visual stimuli, can be tackled. Moreover, a population of neurons learns to extract several independent components if the activity of different neurons are decorrelated by adaptive lateral inhibition. When investigating the mechanisms how learning occurs in our model, we show that IP is necessary for learning, as it enforces a sparse output, guiding learning towards heavy-tailed directions in the input. Lastly, for specific STDP implementations, we show that IP shifts the threshold between potentiation and depression, similar to a sliding threshold for Bienenstock-Cooper-Munro (BCM) learning [22].

The underlying assumption behind our approach, implicit in all standard models of V1 receptive field development, is that both input and output information are encoded in rates. In this light, one may think of our current work as a translation of the model in [16] to a spike-based version. However, the principles behind our model are more general than suggested by our work with rate neurons. We show that the same rule can be applied when inputs are encoded as spike-spike correlation patterns, where a rate-based model would fail.

## Results

**Figure 1:**
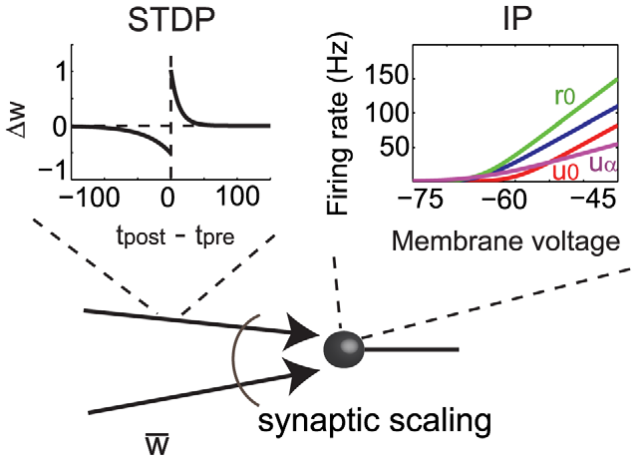
Overview of plasticity rules used for ICA-like learning. Synapse weights 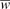 are modified by nearest-neighbor STDP and synaptic scaling. Additionally, intrinsic plasticity changes the neuron's transfer function by adjusting three parameters 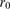, 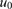, and 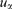. Different transfer functions show the effects of changing each of the three parameters individually relative to the default case depicted in blue. Namely, 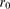 gives the slope of the curve, 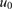 shifts the entire curve left or right, while 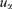 can be used for rescaling the membrane potential axis. Here, 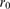 is increased by a factor of 1.5, 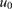 by 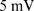, 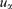 by a factor of 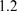.

A schematic view of the learning rules is shown in Fig. 1. The stochastically spiking neuron [23] generates spikes as an inhomogeneous Poisson process, with mean expressed as a function of the total incoming current to the neuron 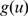, parametrized by variables 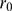, 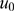 and 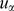. This transfer function is optimized by adapting the three parameters to make the distribution of instantaneous firing rates of the neuron approximatively exponential (for a complete mathematical formulation, see the Methods section). Additionally, Hebbian synaptic plasticity, implemented by nearest-neighbor STDP [24], changes incoming weights and a synaptic scaling mechanism keeps the sum of all incoming weights constant over time.

### A simple demixing problem

To illustrate the basic mechanism behind our approach, we first ask if enforcing a sparse prior by IP and Hebbian learning can yield a valid ICA implementation for the classic problem of demixing two supergaussian independent sources. In the standard form of this problem, zero mean, unit variance inputs ensure that the covariance matrix is the identity, such that simple Hebbian learning with a linear unit (equivalent to principal component analysis) would not be able to exploit the input statistics and would just perform a random walk in the input space. This is, however, a purely mathematical formulation, and does not make much sense in the context of biological neurons. Inputs to real neurons are bounded and —in a rate-based encoding— all positive. Nonetheless, we chose this standard formulation to illustrate the basic underlying principles behind our model. Below, we will consider different spike-based encodings of the input and learning with STDP.

As a special case of a demixing problem, we use two independent Laplacian distributed inputs, with unit variance: 
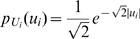
, 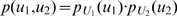. For the linear superposition, we use a rotation matrix 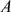:
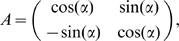
(1)where 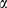 is the angle of rotation, resulting in a set of inputs 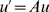. Samples are drawn at each time step from the input distribution and are mapped into a total input to the neuron as 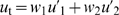, with the weight vector 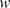 normalized). The neuron's transfer functions 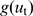, the same as for our spiking model (Fig. 1), is adapted based on our IP rule, to make the distribution of firing rates exponential. For simplicity, here weights change by classic Hebbian learning: 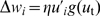, with 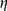 being the synaptic learning rate (see Methods for details). Similar results can be obtained when synaptic changes follow the BCM rule.

**Figure 2:**
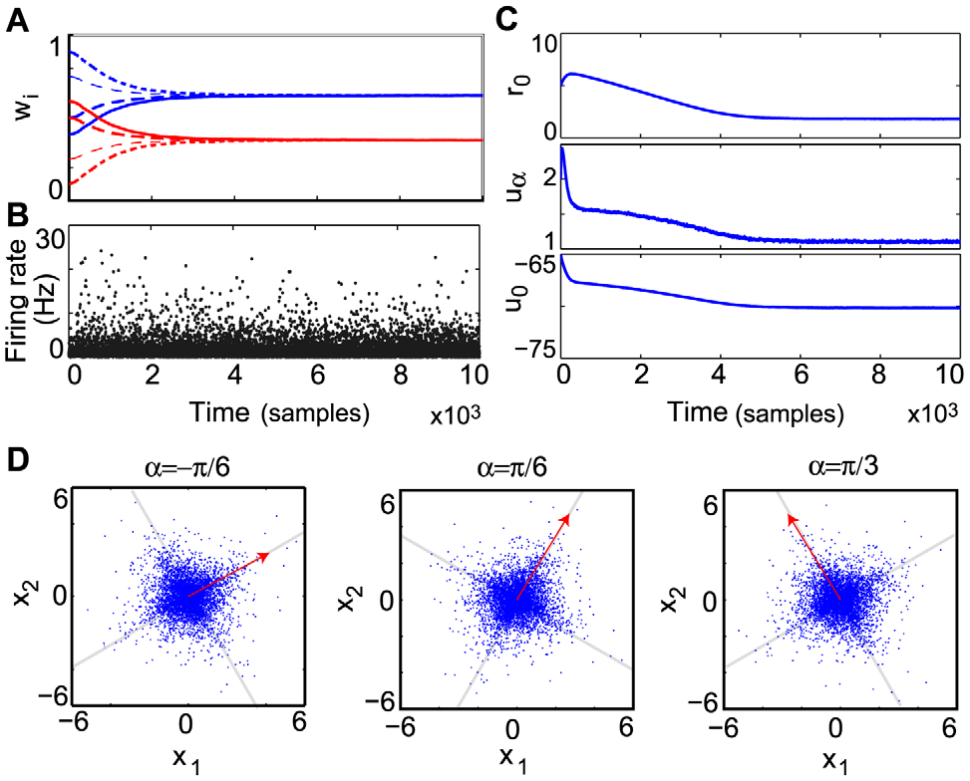
A demixing problem: two rotated Laplace directions. (A) Evolution of the weights (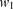 in blue, 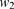 in red) for different initial conditions, with 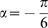, and 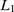 weight normalization. (B) Evolution of the instantaneous firing rate 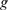, sampled each 1000 ms, for the initial weights 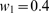, 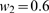. (C) Corresponding changes in transfer function parameters, with 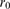 in Hz and 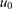 and 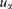 in mV. (D) Final weight vector for different rotation angles 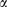 (in red). In the first example, normalization was done by 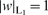 (the estimated rotation angle is 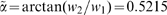, instead of the actual value 0.5236); for the others 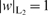 was used. In all cases the final weight vector was scaled by a factor of 5, to improve visibility.

In Fig. 2A we show the evolution of synaptic weights for different starting conditions. As our IP rule adapts the neuron parameters to make the output distribution sparse (Fig. 2B,C), the weight vector aligns itself along the direction of one of the sources. With this simple model, we are able to demix a linear combination of two independent sources for different mixing matrices and different weights constraints (Fig. 2D), as any other single-unit implementation of ICA.

### One neuron learns an independent component

After showing that combining IP and synaptic learning can solve a classical formulation of ICA, we focus on spike-based, biologically plausible inputs. In the following, STDP is used for implementing synaptic learning, while the IP and the synaptic scaling implementations remain the same.

#### Demixing with spikes

The demixing problem above can be solved also in a spike-based setting, after a few changes. First, the positive and negative inputs have to be separated into on- and off- channels (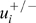) and converted into Poisson spike trains of a certain duration 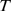 (see Methods), with the corresponding rates 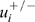 (note that the inputs are no longer white in this four-dimensional space). Secondly, to avoid the unbiological situation of having few very strong inputs, such that single presynaptic spikes always elicit a spike in the postsynaptic neuron, each channel consists of several (here, 25) synapses, with independent inputs having the same firing rate. Synapses are all positive and adapt by STDP, under the normalization 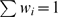. A more detailed description of the parameters can be found in the Methods section.

**Figure 3:**
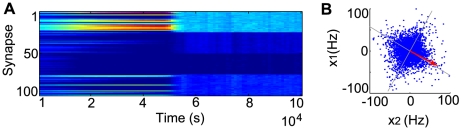
The demixing problem with inputs encoded as spike trains. (A) Evolution of the weights for a rotation angle 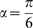. (B) Final corresponding weight vector in the original two-dimensional space. The final weight vector is scaled by a factor of 100, to improve visibility.

The evolution of the weights is shown in Fig. 3A. The corresponding receptive field of the neuron can be obtained by projecting the weight vector back onto the original two-dimensional space (details of this procedure are described in the Methods and Text S1). As in the original formulation of the problem, the neuron receptive field slowly aligns itself along one of the independent components (Fig. 3B).

#### Foldiák's bars: input encoded as firing rates

**Figure 4:**
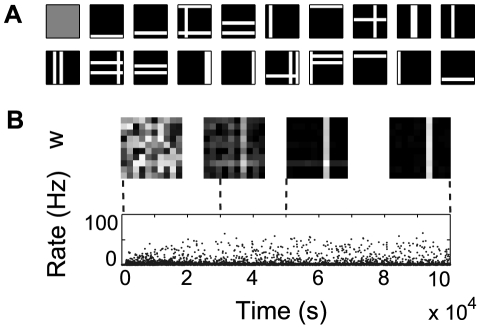
Learning a single independent component for the bars problem. (A) A set of randomly generated samples from the input distribution, (B) Evolution of the neuron's receptive field as the IP rule converges and instantaneous firing rate of the neuron. Each dot corresponds to the instantaneous firing rate (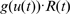) sampled each 500 ms.

As a second test case for our model, we consider Foldiák's well-known bars problem [21]. This is a classic non-linear ICA problem and is interesting from a biological perspective, as it mimics relevant nonlinearities, e.g. occlusions. In the classic formulation, for a two-dimensional input 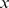, of size 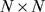, a single bar must be learned after observing samples consisting of the nonlinear superposition of 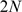 possible individual bars (see Fig. 4A), each appearing independently, with probability 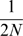 (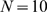). The superposition is non-linear, as the intersection of two bars has the same intensity as the other pixels in the bars (a binary OR).

In our implementation, the input vector is normalized and the value of each pixel 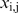 is converted into a corresponding Poisson spike train of duration on the order of a fixation duration [25]. The details of the experimental setup are described in the Methods section. As the IP mechanism begins to take effect, making neuronal activity sparse, the receptive field of the neuron slowly adapts to one of the bars (Fig. 4B). This effect is robust for a wide range of parameters (see Text S2) and, as suggested by our previous results for rate neurons [16], does not critically depend on the particular implementation of the synaptic learning. We obtain similar results with an additive [26] and a simple triplet [27] model of STDP.

The input normalization makes a bar in an input sample containing a single IC stronger than a bar in a sample containing multiple ICs. This may suggest that a single component emerges by preferentially learning ‘easy’ examples, i.e. examples with a single bar. However, this is not the case and one bar is learned even when single bars never appear in the input, as in [28]. Specifically, we use a variant of the bars problem, in which the input always consists of 4 distinct bars, selected at random. In this case, the neuron correctly learns a single component. Moreover, similar results can be obtained for 2–5 bars (see Text S2), using the same set of parameters.

#### Foldiák's bars: input encoded by spike-spike correlations

In the previous experiments, input information was encoded as firing rates. In the following, we show that this stimulus encoding is not critical and the presence of spike-spike correlations is sufficient to learn an independent component, even in the absence of any presynaptic rate modulation. To demonstrate this, we consider a slight modification of the above bars problem, with input samples consisting of always two bars, each two pixel wide, with N distinct bars. This is a slightly more difficult task, as wide bars emphasize non-linearities due to overlap, but can be solved by our model with a rate encoding (see Text S2).

**Figure 5:**
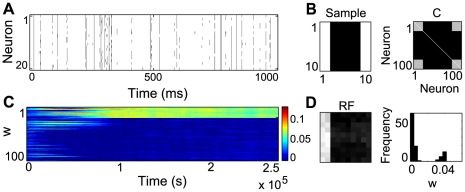
Bars in a correlation-based encoding. (A) Example of 20 spike trains with 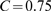. (B) A sample containing two 2-pixel wide bars and the corresponding covariance matrix used for its encoding. (C) Evolution of the weights during learning. (D) Final receptive field and corresponding weights histogram.

In this case, all inputs have the same mean firing rate and the information about whether a pixel belongs to a bar or not is encoded in correlation patterns between different inputs (see Methods). Specifically, background inputs are uncorrelated, while inputs belonging to bars are all pairwise correlated, with a correlation coefficient 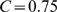 (see Fig. 5B).

As for the rate-based encoding, the neuron is able to reliably learn a bar (Fig. 5C, D). Similar results were obtained for the version with always two bars, each one pixel wide, but with slower convergence. The fact that our approach also works for correlated inputs rests on the properties of STDP and IP. In the original rate formulation, strong inputs lead to higher firing in the postsynaptic neuron, causing the potentiation of the corresponding synapses. Similarly, if presynaptic inputs fire all with the same rate, correlated inputs are more successful in driving the neuron and hence their weights are preferentially strengthened [26]. Moreover, as before, IP enforces sparse post-synaptic responses, guiding learning towards a heavy-tailed direction in the input (a more formal analysis of the interaction between IP and STDP is presented below).

Due to its stochastic nature, our neuron model is not particularly sensitive to input correlations, hence 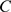 cannot be too small. Stable receptive fields with a single 2 pixel wide bar are still obtained for lower correlation coefficients (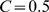), but with slower convergence. We expect better results with a deterministic neuron model, such as the leaky integrate-and-fire. An approximation of IP, based on moment matching could be used in this case. Additionally, STDP-induced competition (due to the relatively small number of inputs, in some cases one weight grows big enough to elicit a spike in the postsynaptic neuron) [26] enforces some constraints on the model parameters to ensure the stability of a solution with multiple non-zero weights. This could be done by increasing the size of the input, restricting the overall mean firing of the neuron 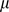 or by slightly changing the STDP parameters (see Methods). These parameter changes do not affect learning with the rate-based encoding, however.

#### Natural scenes input

The third classical ICA problem we consider is the development of V1-like receptive fields for natural scenes [3]. Several computational studies have emphasized that simple cell receptive fields in V1 may be learned from the statistics of natural images by ICA or other similar component extraction algorithms [9], [29], [30]. We hypothesized that the same type of computation could be achieved in our spiking neuron model, by combining different forms of plasticity. Only a rate encoding was used for this problem, partly for computational reasons and partly because it is not immediately obvious how a correlation-based encoding would look like in this case.

We use a set of images from the van Hateren database [31], with standard preprocessing (see Methods). The rectified values of the resulting image patches are linearly mapped into a firing frequency for an on- and off-input population, as done for the bars. The STDP, IP and other simulation parameters are the same as before.

**Figure 6:**
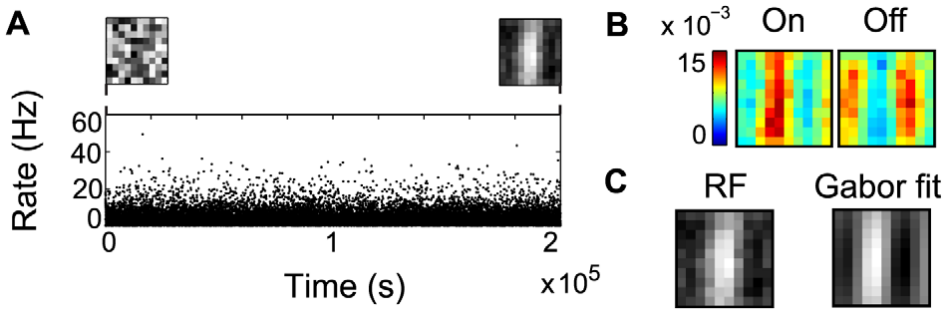
Learning a Gabor-like receptive field. (A) Evolution of the neuronal activity during learning, (B) Learned weights corresponding to the inputs from the on and off populations, (C) The receptive field learned by the neuron, and its l.m.s. Gabor fit.

As shown in Fig. 6A, the receptive field of the neuron computed as the difference between the weights of the on- and the off- input populations (depicted in Fig. 6B) evolves to an oriented filter, similar to those obtained by other ICA learning procedures [9], [29], [30], [32], [33]. A similar receptive field can be obtained by reverse correlation from white noise stimuli. The least-mean-square-error fit to a Gabor wavelet [34] is shown in Fig. 6C. As vertical edges are usually over-represented in the input, the neuron will typically learn a vertical edge filter, with a phase shift depending on the initial conditions. The receptive field has low spatial frequency, but more localized solutions result for a neural population (see below).

### ICA in a neuron population

**Figure 7:**
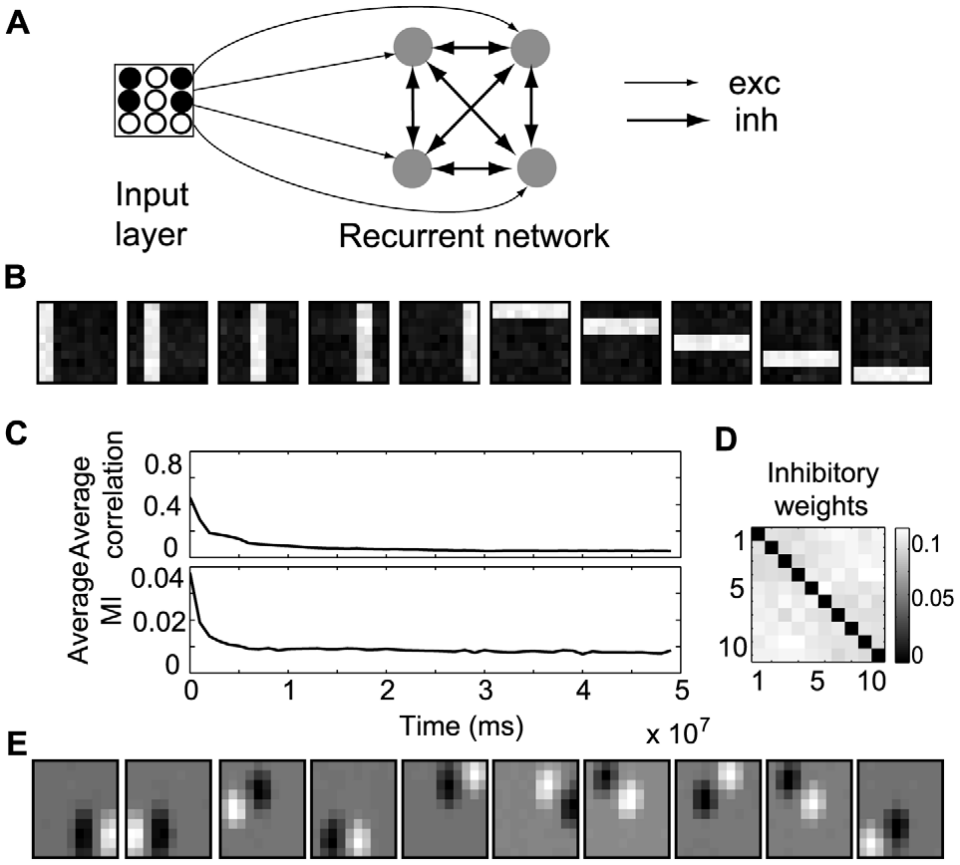
Learning multiple ICs. (A) Overview of the network structure: all neurons receive signals from the input layer and are recurrently connected by all-to-all inhibitory synapses, (B) A set of receptive fields learned for the bars problem, (C) Evolution of the mean correlation coefficient and mutual information in time, both computed by dividing the neuron output in bins of width 1000 s and estimating 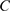 and 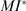 for each bin, (D) Learned inhibitory lateral connections, (E) A set of receptive fields learned for natural image patches.

So far, learning has been restricted to a single neuron. For learning multiple independent components, we implement a neuron population in which the activities of different neurons are decorrelated by adaptive lateral inhibition. This approach is standardly used for feature extraction methods based on single-unit contrast functions [30]. Here, we consider a simple scheme for parallel (symmetrical) decorrelation. The all-to-all inhibitory weights (Fig. 7A) change by STDP and are subject to synaptic scaling, as done for the input synapses. We only use a rate-based encoding for this case, due to computational overhead, which also limits the size of networks we can simulate.

We consider a population of 10 neurons. In order to have a full basis set for the bars problem, we use 2 pixel wide bars. For this case, our learning procedure is able to recover the original basis (Fig. 7B). As lateral inhibition begins to take effect, the average correlation coefficient between the responses of different neurons in the population decreases (Fig. 7C), making the final inhibitory weights unspecific (Fig. 7D). As decorrelation is not a sufficient condition for independence, we show that, simultaneously, the normalized mutual information decreases (see Methods for details). Using the same network for the image patches, we obtain oriented, localized receptive fields (Fig. 7E).

Due to the adaptive nature of IP, the balance between excitation and inhibition does not need to be tightly controlled, allowing for robustness to changes in parameters. However, the inhibition strength influences the time required for convergence (the stronger the inhibition, the longer it takes for the system to reach a stable state). A more important constraint is that the adaptation of inhibitory connections needs to be faster than that of feedforward connections to allow for efficient decorrelation (see Methods for parameters).

### Is IP necessary for learning?

**Figure 8:**
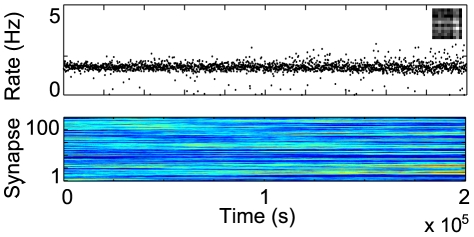
IP is critical for learning. Evolution of the receptive field for a neuron with a fixed gain function, given by the final parameters obtained after learning in the previous bars experiment. A bar cannot be learned in this case.

We wondered what the role of IP is in this learning procedure. Does IP simply find an optimal nonlinearity for the neuron's transfer function, given the input, something that could be computed offline (as for InfoMax [29]), or is the interaction between IP and STDP critical for learning? To answer this question, we go back to Foldiák's bars. We repeat our first bars experiment (Fig. 4) for a fixed gain function, given by the parameters obtained after learning (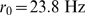, 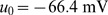, 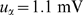). In this case, the receptive field does not evolve to an IC (Fig. 8). This suggests that ICA-like computation relies on the interplay between weight changes and the corresponding readjustment of neuronal excitability, which forces the output to be sparse. Note that this result holds for simulation times significantly larger than in the experiment before, where a bar emerged after 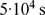, suggesting that, even if the neuron would eventually learn a bar, it would take significantly longer to do so.

**Figure 9:**
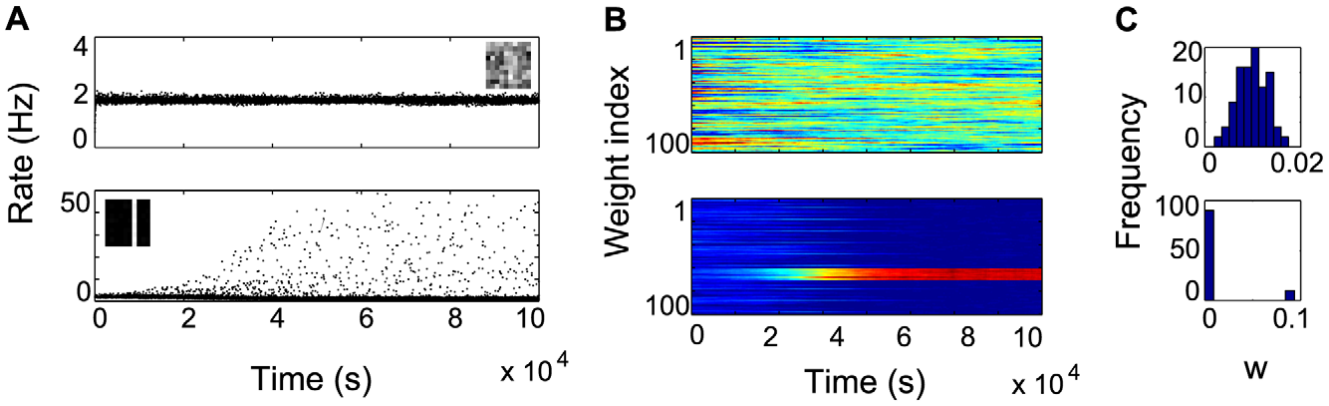
Mean firing constraint is not sufficient for reliable learning. (A) Evolution of neuron activation for a neuron with a gain function regulated by a simplified IP rule, which adjusts 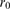 to maintain the same mean average firing 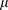. 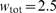 or 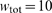, in the first and second row, respectively. Inset illustrates final receptive field for each case. (B) Corresponding evolution of weights and (C) their final distribution.

We could assume that the neuron failed to learn a bar for the fixed transfer function just because the postsynaptic firing was too low, slowing down learning. Hence, it may be that a simpler rule, regulating just the mean firing rate of the neuron, would suffice to learn an IC. To test this hypothesis, we construct an alternative IP rule, which adjusts just 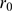 to preserve the average firing rate of the neuron (see Methods). With the same setup as before and the new IP rule, no bar is learned and the output distribution is Gaussian, with a small standard deviation around the target value 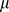 (Fig. 9A). However, after additional parameter tuning, a bar can sometimes be learned, as shown in Fig. 9B. In this case, the final output distribution is highly kurtotic, due to the receptive field. The outcome depends on the variance of the total input, which has to be large enough to start the learning process (variance was regulated by the parameter 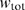, see Methods). Most importantly, this dependence on model parameters shows that regulating the mean of the output distribution is not sufficient for reliably learning a bar and higher order moments need to be considered as well.

### Interaction between IP and STDP

A good starting point for elucidating the mechanism by which the interaction between STDP and IP facilitates the discovery of an independent component is our initial problem of a single unit receiving a two dimensional input. We have previously shown in simulations that for a bounded, whitened, two dimensional input the weight vector tends to rotate towards the heavy-tailed direction in the input [16]. Here, we extend these results both analytically and in simulations. Our analysis focuses on the theoretical formulation of zero mean, unit variance inputs used for the demixing problem before and is restricted to expected changes in weights given the input and output firing rates, ignoring the time of individual spikes.

We report here only the main results of these experiments, while a detailed description is provided as supplemental information (see Text S3). Firstly, for conveniently selected pairs of input distributions, it is possible to show analytically that the weight vector rotates towards the heavy-tailed direction in the input, under the assumption that IP adaptation is faster than synaptic learning (previously demonstrated numerically in [16]). Secondly, due to the IP rule, weight changes mostly occur on the tail of the output distribution and are significantly larger for the heavy-tailed input. Namely, IP focuses learning to the heavy tailed direction in the input. When several inputs are supergaussian, the learning procedure results in the maximization of the output kurtosis, independent of the shape of the input distributions. Most importantly, we show that, for simple problems when a solution can be obtained by nonlinear PCA, our IP rule significantly speeds up learning of an independent component.

One way to understand these results could be in terms of nonlinear PCA theory. Given that for a random initial weight vector, the total input distribution is close to Gaussian, in order to enforce a sparse output, the IP has to change the transfer function in a way that ‘hides’ most of the input distribution (for example by shifting 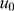 somewhere above the mean of the Gaussian). As a result, the nonlinear part of the transfer function will cover the ‘visible’ part of the input distribution, facilitating the discovery of sparse inputs by a mechanism similar to nonlinear PCA. In this light, IP provides the means to adapt the transfer function in a way that makes the nonlinear PCA particularly efficient.

Lastly, from an information-theoretic perspective, our approach can be linked to previous work on maximizing information transmission between neuronal input and output by optimizing synaptic learning [23]. This synaptic optimization procedure was shown to yield a generalization of the classic BCM rule [22]. We can show that, for a specific family of STDP implementations, which have a quadratic dependence on postsynaptic firing, IP effectively acts as a sliding threshold for BCM learning (see Text S4).

## Discussion

Although ICA and related sparse coding models have been very successful in describing sensory coding in the cortex, it has been unclear how such computations can be realized in networks of spiking neurons in a biologically plausible fashion. We have presented a network of stochastically spiking neurons that performs ICA-like learning by combining different forms of plasticity. Although this is not the only attempt at computing ICA with spiking neurons, in previous models synaptic changes were not local, depending on the activity of neighboring neurons within a population [6], [7]. In this light, our model is, to our knowledge, the first to offer a mechanistic explanation of how ICA-like computation could arise by biologically plausible learning mechanisms.

In our model, IP, STDP and synaptic scaling interact to give rise to robust receptive field development. This effect does not depend on a particular implementation of STDP, but it does require an IP mechanism which enforces a sparse output distribution. Although there are very good theoretical arguments why this should be the case [11], [13], [16], the experimental evidence supporting this assumption is limited [12]. A likely explanation for this situation is the fact that it is difficult to map the experimentally observable output spikes into a probability of firing. Spike count estimates cannot be used directly, as they critically depend on the bin size. Additionally, the inter-spike interval (ISI) of an inhomogeneous Poisson process with exponentially distributed mean 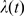 is indistinguishable from the ISI of a homogeneous Poisson distribution with mean 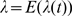. Hence, more complex statistical analyses are required for disentangling the two (see [35]).

From a computational perspective, our approach is reminiscent of several by-now classic ICA algorithms. As mentioned before, IP enforces the output distribution to be heavy-tailed, like in sparse coding [9]. Our model also shares conceptual similarities to InfoMax [29], which attempts to maximize output entropy (however, at the population level) by regulating the weights and a neuron threshold parameter. Maximizing information transmission between pre- and post-synaptic spike trains under the constraint of a fixed mean postsynaptic firing rate links our method to previous work on synaptic plasticity. A spike-based synaptic rule optimizing the above criterion [23] yields a generalization of the BCM rule [22], a powerful form of learning, which is able to discover heavy-tailed directions in the input [36], [37] and to learn Gabor receptive fields [38] in linear neurons. We have shown that, sliding threshold BCM can be viewed as a particular case of IP learning, for a specific family of STDP models.

It is interesting to think of the mechanism presented here in relation to projection pursuit [39], which tries to find good representations of high-dimensional spaces by projecting data on a lower dimensional space. The algorithm searches for interesting projection directions, a typical measure of interest being the non-Gaussianity of the distribution of data in the lower dimensional space. The difference here is that, although we do not explicitly define a contrast function maximizing kurtosis or other similar measure, our IP rule implicitly yields highly kurtotic output distributions. By sparsifying the neuron output, IP guides the synaptic learning towards the interesting (i.e. heavy-tailed) directions in the input.

From a different perspective, we can relate our method to nonlinear PCA. It is known that, for zero mean whitened data, nonlinear Hebbian learning in a rate neuron can successfully capture higher order correlations in the input [40], [41]. Moreover, it has been suggested that the precise shape of the Hebbian nonlinearity can be used for optimization purposes, for example for incorporating prior knowledge about the sources' distribution [40]. IP goes one step further in this direction, by adapting the transfer function online, during learning. From a biological perspective, there are some advantages in adapting the neuron's excitability during computation. Firstly, IP speeds up the nonlinear decorrelation of inputs. Secondly, the system gains great robustness to changes in parameters (as demonstrated in Text S2). Additionally, IP regulation plays a homeostatic role, making constraints on the input mean or second order statistics unnecessary. In the end, all the methods we have mentioned are closely related and, though conceptually similar, our approach is another distinct solution.

Our previous work was restricted to the a rate model neuron [16]. Beyond translating our results to a spiking neuron model, we have shown here that similar principles can be applied when information is encoded as spike-spike correlations, where a model relying just on firing rates would fail. It is a interesting challenge for future work to further investigate the exact mechanisms of receptive field development for different types of input encoding.

## Methods

### Neuron model

We consider a stochastically spiking neuron with refractoriness [23]. The model defines the neuron's instantaneous probability of firing as a function of the current membrane potential and the refractory state of the neuron,which depends on the time since its last spike. More specifically, the membrane potential is computed as 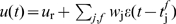 where 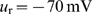 is the resting potential, while the second term represents the total incoming drive to the neuron, computed as the linear summation of post-synaptic potentials evoked by incoming spikes. Here, 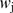 gives the strength of synapse 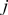, 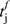 is the time of a presynaptic spike, and 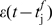 the corresponding evoked post-synaptic potential, modeled as a decaying exponential, with time constant 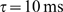 (for GABA-ergic synapses, 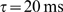) and amplitude 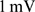.

The refractory state of the neuron, with values in the interval 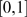, is defined as a function of the time of the last spike 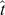, namely:
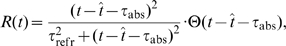
where 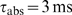 gives the absolute refractory period, 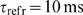 is the relative refractory period and 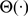 is the Heaviside function.

The probability 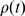 of the stochastic neuron firing at time 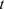 is given as a function of its membrane potential and refractory state [23] 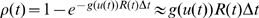 where 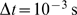 is the time step of integration, and 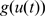 is a gain function, defined as: 
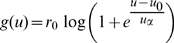
 Here 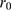 , 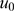 and 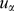 are model parameters, whose values are adjusted by intrinsic plasticity, as described below.

### Intrinsic plasticity

Our intrinsic plasticity model attempts to maximize the mutual information between input and output, for a fixed energy budget [16], [42]. More specifically, it induces changes in neuronal excitability that lead to an exponential distribution of the instantaneous firing rate of the neuron [13]. The specific shape of the output distribution is justified from an information theoretic perspective, as the exponential distribution has maximum entropy for a fixed mean. This is true for distributions defined on the interval 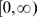, but, under certain assumptions, can be a good approximation for the case where the interval is bounded, as it happens in our model due to the neuron's refractory period (see below). Optimizing information transmission under the constraint of a fixed mean is equivalent to minimizing the Kullback-Leibler divergence between the neuron's firing rate distribution and that of an exponential with mean 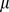:
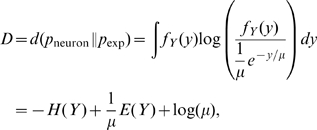
with 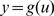 and 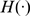 denoting the entropy and 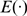 the expected value. Note that the above expression assumes that the instantaneous firing rate of the neuron is proportional to 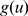, that is that 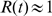. When taking into account the refractory period of the neuron, which imposes an upper-bound 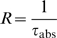 on the output firing rate, the maximum entropy distribution for a specific mean 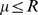 is a truncated exponential [43]. The deviation between the optimal exponential for the infinite and the bounded case depends on the values of 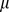 and 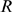, but it is small in cases in which 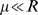. Hence, our approximation is valid as long as the instantaneous firing rate 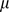 is significantly lower than 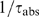, that is when the mean firing rate of the neuron is small. In our case, we restrict 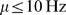. If not otherwise stated, all simulations have 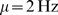. Note also that the values considered here are in the range of firing rates reported for V1 neurons [44].

Computing the gradient of 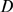 for 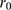, 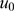 and 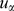, and using stochastic gradient descent, the optimization process translates into the following update rules [42]:
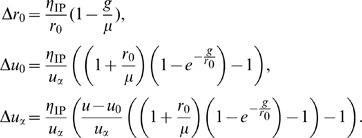
Here, 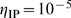 is a small learning rate. Here, the instantaneous firing rate is assumed to be directly accessible for learning. Alternatively, it could be estimated based on the recent spike history.

Additionally, as a control, we have considered a simplified rule, which adjusts a single transfer function parameter in order to maintain the mean firing rate of the neuron to a constant value 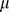. More specifically, a low-pass-filtered version of the neuron firing rate is used to estimate the current mean firing rate of the neuron 
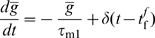
 where 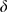 is the Dirac function and 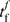 is the time of firing of the post-synaptic neuron and 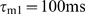. Based on this estimate, the value of the parameter 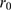 is adjusted as 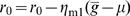 Here, 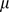 is the goal mean firing rate, as before and 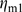 is a learning rate, set such that, for a fixed Gaussian input distribution, convergence is reached as fast as for our IP rule described before (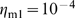).

### Synaptic learning

The STDP rule implemented here considers only nearest-neighbor interactions between spikes [24]. The change in weights is determined by:
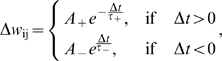
where 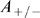 is the amplitude of the STDP change for potentiation and depression, respectively (default values 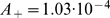 and 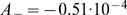), 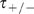 are the time scales for potentiation and depression (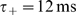, 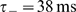; for learning spike-spike correlations 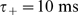)[19], and 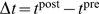 is the time difference between the firing of the pre- and post-synaptic neuron. For the lateral inhibitory connections, the STDP learning is faster, namely 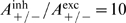. In all cases, weights are always positive and clipped to zero if they become negative.

This STDP implementation is particularly interesting as it can be shown that, under the assumption of uncorrelated or weakly correlated pre- and post-synaptic Poisson spike trains, it induces weight changes similar to a BCM rule, namely [24]:
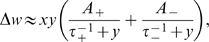
where 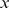 and 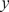 are the firing rates of the pre- and post-synaptic neuron, respectively.

For the above expression, the fixed BCM threshold can be computed as:
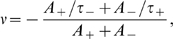
which is positive when potentiation dominates depression on the short time scale, while, overall, synaptic weakening is larger than potentiation:
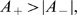


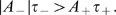



In some experiments we also consider the classical case of additive all-to-all STDP [26], which acts as simple Hebbian learning, the induced change in weight being proportional to the product of the pre- and post-synaptic firing rates (see [24] for comparison of different STDP implementations). The parameters used in this case are: 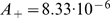, 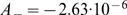 and the same time constants as for the nearest neighbor case. Additionally, the simple triplets STDP model used as an alternative BCM-like STDP implementation is described in Text S4.

### Synaptic scaling

As in approaches which directly maximize kurtosis or similar measures [16], [30], [40], the weight vector is normalized: 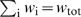, with 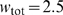, with weights being always positive. This value is arbitrary, as it represents a scaling factor of the total current, which can be compensated for by IP. It was selected in order to keep the final parameters close to those in [23]. Additionally, for the natural image patches the normalization was done independently for the on- and off- populations, using the same value for 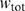 in each case.

In a neural population, the same normalization is applied for the lateral inhibitory connections. As before, weights do not change sign and are constrained by the 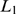 norm: 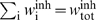, with 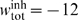.

Currently, the normalization is achieved by dividing each weight by 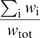, after the presentation of each sample. Biologically, this operation would be implemented by a synaptic scaling mechanism, which multiplicatively scales the synaptic weights to preserve the average input drive received by the neuron [20].

### Setup for experiments

In all experiments, excitatory weights were initialized at random from the uniform distribution and normalized as described before. The transfer function was initialized to the parameters in [23] (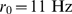, 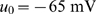, 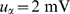). Unless otherwise specified, all model parameters had the default values defined in the corresponding sections above. For all spike-based experiments, each sample was presented for a time interval 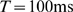, followed by the weight normalization.

For the experiments involving the rate-based model and a two-dimensional input, each sample was presented for one time step and the learning rates for IP and Hebbian learning were 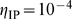 and 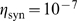, respectively. In this case, the weight normalization procedure can influence the final solution. Namely, positive weights with constant 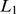 norm always yield a weight vector in the first quadrant, but this limitation can be removed by a different normalization, which keeps the 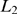 norm of the vector constant (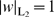).

For the demixing problem, the input was generated as described for the rate-based scenario above. After the rectification, the firing rates of the input on the on- and off- channels were scaled by a factor of 20, to speed up learning. After convergence, the total weight of each channel was estimated as the sum of individual weights corresponding to that input. The resulting four-dimensional weight vector was projected back to the original two-dimensional input space using: 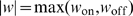, with a sign given by that of the channel with maximum weight (positive for 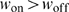, negative otherwise). This procedure results by a minimum error projection of the weight vector onto the subspace defined by the constraint 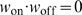, see Text S1 for details.

For all variants of the bars problem, the input vector was normalized to 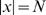, with 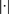 defining the 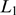 norm, as in [45]. Inputs were encoded using firing rates with mean 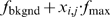, where 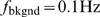 is the frequency of a background pixel and 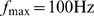 gives the maximum input frequency, corresponding to a sample containing a single bar in the original bars problem.

When using the correlation-based encoding, all inputs had the same mean firing rate (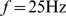). Inputs corresponding to pixels in the background were uncorrelated, while inputs belonging to bars were all pairwise correlated, with a correlation coefficient 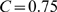. Poisson processes with such correlation structure can be generated in a computationally efficient fashion by using dichotomous Gaussian distributions [46].

When learning Gabor receptive fields, images from the van Hateren database [31] were convolved with a difference-of-gaussians filter with center and surround widths of 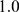 and 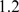 pixels, respectively. Random patches of size 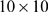 were selected from various positions in the images. Patches having very low contrast were discarded. The individual input patches were normalized to zero mean and unit variance, similar to the processing in [45]. The rectified values of the resulting image were mapped into a firing frequency for an on- and off-input population (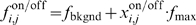) and, as before, samples were presented for a duration 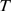.

For a neuronal population, input-related parameters were as for the single component, but with 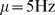, to speed up learning. The initial parameters of the neuron transfer function were uniformly distributed around the default values mentioned above, with variance 0.1, 5, and 0.2 for 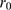, 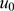, and 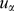, respectively. Additionally, the inhibitory weights were initialized at random, with no self-connections, and normalized as described before. The mutual information (MI), estimated within a window of 1000 s, was computed as 
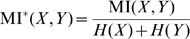
, with 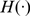 denoting the entropy (see [45]), applied for the average firing rate of the neurons for each input sample.

## Supporting Information

Text S1 Receptive field estimation for the spike-based demixing problem
(0.04 MB PDF)

Text S2 Parameter dependence for learning one IC
(0.07 MB PDF)

Text S3 Learning with a two-dimensional input
(0.53 MB PDF)

Text S4 A link to BCM
(0.15 MB PDF)
