# Supplementary material for: Independent Component Analysis in Spiking Neurons
Source: PLoS Comput Biol. 2010 Apr 22;6(4):e1000757. doi: 10.1371/journal.pcbi.1000757 (PMC2858697; doi:10.1371/journal.pcbi.1000757)
Supplement: Text S1 — Receptive field estimation for the spike-based demixing problem (0.04 MB PDF) [file pcbi.1000757.s001.pdf]

S1. Receptive field estimation for the spike-based demixing problem

In order to recover the receptive field of the neuron after learning, we need to project the final weight vector back to the subspace defined by the input. Given that inputs can either be positive or negative but not both, this subspace can be easily defined by the constraint $w_i^{\mathrm{on}} \cdot w_i^{\mathrm{off}} = 0$, where the index i refers to the different input dimensions, and takes here the values 1 or 2. Hence, the problem of recovering the receptive field of the neuron can be cast as a constraint optimization problem: we want to find the vector $\tilde{w} = (\tilde{w}_1^{\mathrm{on}}, \tilde{w}_1^{\mathrm{off}}, \tilde{w}_2^{\mathrm{on}}, \tilde{w}_2^{\mathrm{off}})$ from the subspace defined by the constraint above, such that the distance between $\tilde{w}$ and the original vector w is minimized. For simplicity, we consider here the Euclidean distance as metric. Our cost function is then:

$$D(\tilde{w}) = \sum \left[(\tilde{w}_i^{\mathrm{on}} - w_i^{\mathrm{on}})^2 + (\tilde{w}_i^{\mathrm{off}} - w_i^{\mathrm{off}})^2 \right],$$

which needs to be minimized under the constraints $\tilde{w}_i^{\mathrm{on}} \cdot \tilde{w}_i^{\mathrm{off}} = 0$, with $i = \{1, 2\}$.

This problem can be solved using Lagrange multipliers. Namely, we define an alternative cost function:

$$L(\tilde{w}) = \sum_i \left[(\tilde{w}_i^{\mathrm{on}} - w_i^{\mathrm{on}})^2 + (\tilde{w}_i^{\mathrm{off}} - w_i^{\mathrm{off}})^2 \right] + \sum_i \lambda_i \tilde{w}_i^{\mathrm{on}} \cdot \tilde{w}_i^{\mathrm{off}}.$$

When computing the derivative of L, we can easily see that each pair of on- and off- weights give an independent system of equations, with identical form:

$$\begin{cases} 2 \cdot (\tilde{w}_i^{\mathrm{on}} - w_i^{\mathrm{on}}) + \lambda_i \cdot \tilde{w}_i^{\mathrm{off}} & = & 0 \\ 2 \cdot \left(\tilde{w}_i^{\mathrm{off}} - w_i^{\mathrm{off}}\right) + \lambda_i \cdot \tilde{w}_i^{\mathrm{on}} & = & 0 \\ \tilde{w}_i^{\mathrm{on}} \cdot \tilde{w}_i^{\mathrm{off}} & = & 0. \end{cases}$$

By solving the above system for each dimension, we obtain solutions of the form: $\tilde{w}_i^{\mathrm{on}} = w_i^{\mathrm{on}}$ and $\tilde{w}_i^{\mathrm{off}} = 0$, or alternatively $\tilde{w}_i^{\mathrm{off}} = w_i^{\mathrm{off}}$ and $\tilde{w}_i^{\mathrm{on}} = 0$. Both solutions are extrema of the cost function L, but only one is our searched minimum. A quick check of the nature of each extremum shows that the final solution corresponds to the larger of the two weights. This means that the receptive field of the neuron can be computed by $|w_i| = \max\{w_i^{\mathrm{on}}, w_i^{\mathrm{off}}\}$, with the sign given by $\mathrm{argmax}\{\mathrm{w_i^{on}}, \mathrm{w_i^{off}}\}$, i.e. positive if $w_i^{\mathrm{on}} > w_i^{\mathrm{off}}$, negative otherwise.
