# Supplementary material for: Independent Component Analysis in Spiking Neurons
Source: PLoS Comput Biol. 2010 Apr 22;6(4):e1000757. doi: 10.1371/journal.pcbi.1000757 (PMC2858697; doi:10.1371/journal.pcbi.1000757)
Supplement: Text S2 — Parameter dependence for learning one IC (0.07 MB PDF) [file pcbi.1000757.s002.pdf]

S2. Parameter dependence for learning one IC

The proposed IC learning model is extremely robust to changes in a variety of parameters. In the case of the bars problem, for example, the neuron will always learn a bar, independent of initial conditions (w_{tot}, initial transfer function parameters r_0, u_0 and u_α), the IP-enforced mean firing rate ($\mu \in [1, 15]$ Hz), variations in the learning rate for IP ($\eta \in [10^{-8},\ 10^{-4}]$), the duration of a stimulus presentation ($T \in [50, 500]$ms), the STDP parameters (the threshold between potentiation and depression ν, varied by changing the ratio A_+/A_- or one of the time constants $\tau_\pm$, withing the range $\nu \in [0.1, 15]$, see Methods), or variations in the average probability of a bar ($[1/2N, 1/N]$). Moreover, similar receptive fields can be obtained with different synaptic plasticity rules, such as additive [1] or simple triplet [2] STDP.

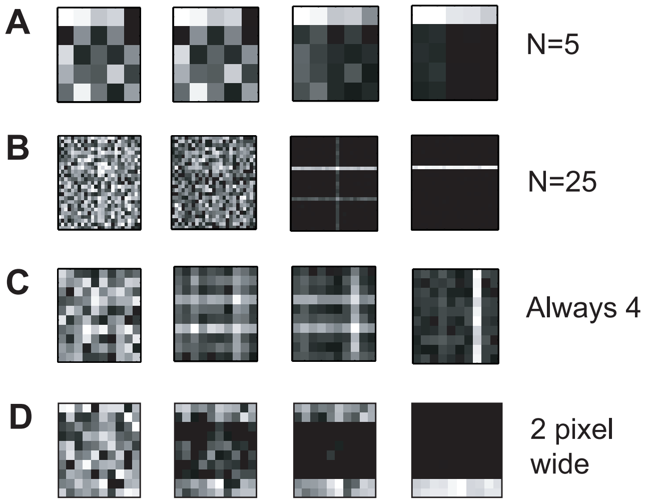

Figure 1. Learning an IC. (A) and (B) The original bars problem, with different input sizes $N = 5$ and $N = 25$, respectively, (C) Modified bars problem, in which each sample consists of exactly 4 superimposed bars, (D) Modified bars problem, in which bars are two pixel wide and each sample consists of exactly 2 superimposed bars. Except for the varied variable (N), all model parameters are fixed to the set of default values described in the Methods.

As seen in Fig. 1A and B, a single bar is learned for different input sizes (5 to 25) for the original bars problem. Moreover, the rule handles equally well more difficult variants of the bars problem [3], in which samples consist always of the same number of bars (samples containing 2-5 bars yield good results, e.g. 4 bars in Fig. 1C), or in which bars that are two-pixel wide (Fig. 1D), emphasizing the nonlinearity of the superposition. However, samples containing many bars tend to converge somewhat slower (approximatively by a factor of 2 for always 4 bars), corresponding to a lower input frequency (due to our input normalization procedure).

References

1. Song S, Miller K, Abbott L (2000) Competitive Hebbian learning through spike-timing-dependent synaptic plasticity. Nature Neurosci 3: 919-926.

2. Pfister J, Gerstner W (2006) Triplets of spikes in a model of spike timing-dependent plasticity. Journal of Neuroscience 26: 9673-9682.

3. Lücke J, Sahani M (2008) Maximal causes for non-linear component extraction. Journal of Machine Learning Research 9: 1227-1267.
