# Supplementary material for: Independent Component Analysis in Spiking Neurons
Source: PLoS Comput Biol. 2010 Apr 22;6(4):e1000757. doi: 10.1371/journal.pcbi.1000757 (PMC2858697; doi:10.1371/journal.pcbi.1000757)
Supplement: Text S3 — Learning with a two-dimensional input (0.53 MB PDF) [file pcbi.1000757.s003.pdf]

S3. Learning with a two-dimensional input

The weight vector rotates toward the heavy-tailed direction in the input

For elucidating the mechanism by which the interaction between STDP and IP may facilitate the discovery of an independent component, we go back to the simplified problem of a single unit receiving a two dimensional input. This analysis is restricted to the theoretical formulation of zero mean, unit variance inputs and does not consider the time of individual spikes. Additionally, for mathematical convenience, the rotational angle α is zero in this case. Previously, we have shown in simulations that for a bounded, whitened, two dimensional input the weight vector tends to rotate towards the heavy-tailed direction in the input [1]. Here, we show analytically that the same result holds for the zero mean, unit variance uniform and the standard Cauchy distribution (see Fig. 1A). The two inputs are independent ($p(u_1, u_2) = p_{U_1}(u_1) \cdot p_{U_2}(u_2)$), with the PDFs defined by:

$$\begin{aligned} p_{U_1}(u_1) &= \begin{cases} \frac{1}{2\sqrt{3}}, & \text{for} |u_1| \leq \sqrt{3} \\ 0, & \text{otherwise} \end{cases}, \\ p_{U_2}(u_2) &= \frac{1}{\pi(1+u_2^2)}. \end{aligned}$$

As before, a linear relation is assumed between the input and the induced changes of voltage in the postsynaptic neuron. Additionally, the IP adaptation is taken to be much faster than synaptic plasticity [1] for the analytical derivation.

Under the above assumptions, given the two inputs and the corresponding weight vector (w_1, w_2), the probability distribution for the total input u of the neuron ($u = u_1 + u_2$) can be computed as a convolution of the probability distributions of u_1 and u_2. For simplicity, we only consider here a weight vector along the first diagonal, but the analysis could be extended to any other weight vector. For this case, the result of the convolution is:

$$p_{U=U1+U2}(u) = p_{U_1}(u_1) * p_{U_2}(u_2) = \frac{1}{2\sqrt{3}\pi} \left(\arctan(u_+) - \arctan(u_-) \right),$$

where * marks the convolution operator and $u_{+/-} = u \pm \sqrt{3}$ (see Fig. 1B).

Under the assumption of a fast IP, the neuron's gain becomes close to the optimal function $\tilde{g}$, while the weights remain approximately unchanged. Knowing the input distribution, $\tilde{g}$ can be computed analytically. Specifically, we exploit the standard relation $p_G(g) = \left(\frac{\mathrm{d}g}{\mathrm{d}u}\right)^{-1} p_U(u)$ and the constraint that the output distribution should be exponential. By integrating the above equation, with p_U as computed before and $p_G(g) = \frac{1}{\mu} e^{-\frac{g}{\mu}}$, we obtain the optimal transfer function (see Fig. 1C):

$$\tilde{g} = -\mu \log \left(\frac{1}{2} + \frac{1}{2\sqrt{3}\pi} \left(u_- \arctan(u_-) - u_+ \arctan(u_+) + \frac{1}{2} \log \left(\frac{1+u_+^2}{1+u_-^2} \right) \right) \right).$$

.

Knowing $\tilde{g}$, the estimated change in weights by classic Hebbian learning, under the assumption that $E(R(t)) \approx 1$, can be computed as:

$$\begin{aligned} \Delta w_1 &= E(u_1 g) = \int x \tilde{g}(y) p_{U_1}(x) p_{U_2}(y-x) \mathrm{d}x \mathrm{d}y, \\ \Delta w_2 &= E(u_2 g) = \int (y-x) \tilde{g}(y) p_{U_1}(x) p_{U_2}(y-x) \mathrm{d}x \mathrm{d}y. \end{aligned}$$

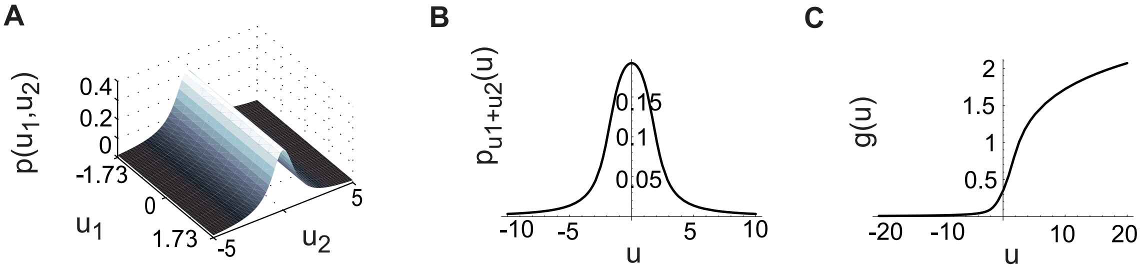

Figure 1. Mathematical analysis for a two-dimensional input space (A) The joint probability distribution of the input, (B) Distribution of the total synaptic current, given the total input for a weight vector along the first diagonal, (C) Corresponding optimal neuronal gain function.

Integrating the above relations, we obtain that $\Delta w_1 < \Delta w_2$, meaning that the learning procedure rotates the weight vector towards the heavy-tailed direction in the input. Similar results can be obtained numerically for a rate-based equivalent of our STDP rule (see Methods). Intuitively, the largest changes in weight occur for large neuron responses, which corresponds to samples from the right tail of the input distribution. As the uniform distribution has only finite support, here the output tail is determined by the other input. These results apply to any IP rule that enforces an exponential output distribution. In practice, implementations corresponding to different families of transfer functions may work more or less well, depending on the specific problem (i.e. on how well $\tilde{g}$ can be approximated).

Most learning occurs on the tail of the output distribution

To build more intuition, and to show that our analytical results hold also for distributions with infinite support, we repeat the above experiment for a Gaussian and a Laplacian distribution:

$$\begin{aligned} p_{U_1}(u_1) &= \frac{1}{\sqrt{2\pi}} e^{-\frac{u_1^2}{2}}, \\ p_{U1}(u_2) &= \frac{1}{\sqrt{2}} e^{-\sqrt{2}|u_2|}. \end{aligned}$$

As before, the PDF for the total input is $p_U(u) = p_{U_1}(u_1) * p_{U_2}(u_2)$, which, unfortunately, does not have a closed-form solution in this case. However, as in [1], we can generate samples from the distribution and obtain an estimate of the optimal transfer function by our IP rule. For the approximated optimal parameters $\tilde{r}_0$ $\tilde{u}_0$ $\tilde{u}_\alpha$, we can again estimate the changes in weights with nearest-neighbor STDP (see [2] and Methods) as:

$$\Delta w_{\mathrm{i}} \propto u_{\mathrm{i}} \tilde{g}(u) \left(\frac{A_+}{\tau_+^{-1} + \tilde{R} g(u)} + \frac{A_-}{\tau_-^{-1} + \tilde{r} g(u)} \right), \tag{1}$$

with $u = w_1 u_1 + w_2 u_2$, $i = 1, 2$. The proportionality factor is given by the learning rate of the STDP rule and by the average value of the refractory state of the neuron $R(t)$ (we consider again $\tilde{R} \approx 1$).

In Fig. 2, we plot the change in weights for different input samples, ordered by the corresponding neuron activation. One can easily note that significant changes in weights occur only on the tail of the output distribution, as expected by a Hebbian rule. Importantly, for the same output, weight changes are much larger for the heavy-tailed directions in the input, suggesting that IP guides weight towards the heavy-tailed direction in the input. Moreover, this property does not depend on the initial state of the weight vector (except for the extreme case where $w_1 \gg w_2$, where the gradient becomes very flat, making it difficult for the IP rule to converge). It holds also when weights change by classic Hebbian

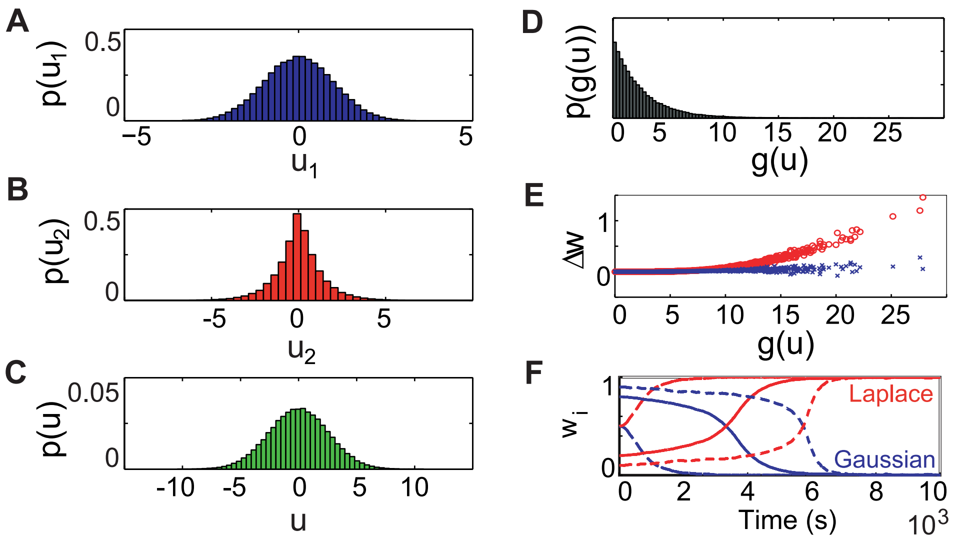

Figure 2. A two dimensional input space, both directions with infinite support. (A) and (B) Probability distributions for the two independent directions, (C) The distribution of total input for $w = (0.6, 0.4)$, (D) Output distribution of the neuron, after the IP rule has converged, (E) Change in weights for the Gaussian (u_1, blue crosses) and Laplacian (u_1 red circles) directions, ordered by the instantaneous firing rate of the output neuron, (F) The actual evolution of the weight vector when the IP and STDP rule are acting in parallel (w_1 in blue, w_2 in red, different dashes show variations in initial conditions). The weight vector aligns with the heavy-tailed direction u_2, independent of initial conditions.

learning and when inputs are mixed using a matrix A, as described in the main text for two Laplacian directions. Consequently, when the STDP and IP rules are acting together, the neuron will learn to respond preferentially to the Laplace direction (u_2) in the input, independent of the initial value of w (Fig. 2F).

Learning maximizes kurtosis

In the case of multiple heavy-tails in the input, it is expected that ICA would select one single such direction, as we have demonstrated for the demixing problem with a two-dimensional input. This can be explained mathematically by the fact that the kurtosis of a linear mixture of several non-Gaussian sources is always smaller than that of each of its components [3]. Hence, maximizing kurtosis—a very common objective for ICA—means reducing the weighted sum of several directions to a single direction, by making all but one of the weights equal to zero. It is easy to show that the same principle applies to our rule (Fig. 3A), for two independent Laplace inputs. Depending on the initial conditions, the neuron will develop sensitivity to one of the two directions. When the initial weights are equal (the solid lines), the winning input is selected at random.

Although the shape of the output distribution enforced by IP may suggest a bias for finding exponential tails in the input, we found that our rule generally selects the heaviest tail distribution in the input. Specifically, when the input consists of a Laplace and a Cauchy distribution, the latter is preferred, as required in ICA (see Fig. 3B).

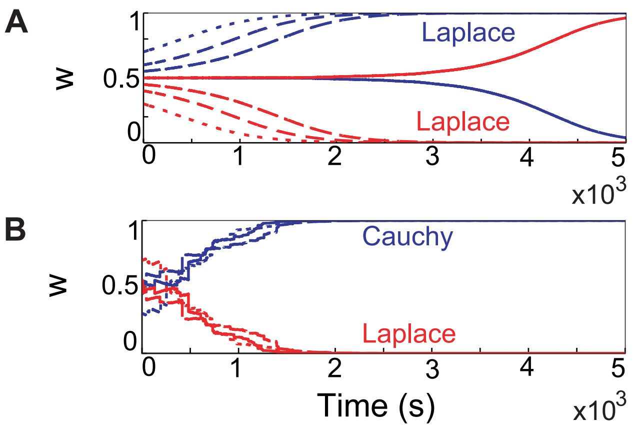

Figure 3. Maximizing the kurtosis of the output by IP. (A) Evolution of the weight vector when the IP and STDP rule are acting in parallel for the case of two Laplacian inputs. As function of initial conditions, the learned receptive field prefers one of the directions, maximizing kurtosis. (B) In the case of a Cauchy (red) and Laplace (blue) distribution, the more heavy-tailed direction is preferred.

IP speeds up learning

We have shown in the main text that a bar cannot be reliably learned with a fixed transfer function. However, for a linear superposition of independent sources, theoretical studies suggest that —with some preprocessing and an appropriate fixed nonlinearity— a receptive field could develop by nonlinear PCA [4] in a rate neuron model. Even for the linear superposition of inputs, adapting the transfer function online may be advantageous, e.g. in terms of convergence time. To analyze this, we consider again the problem of learning the heavy tail direction in a two-dimensional input, with a Gaussian and Laplacian component, normalized to zero mean and unit variance. Although in this simplified case a fixed nonlinearity is sufficient for discovering the heavy-tailed direction, as predicted, learning is faster in the case when IP interacts with synaptic learning, compared to the case when the final transfer function obtained after learning is used (Fig. 4).

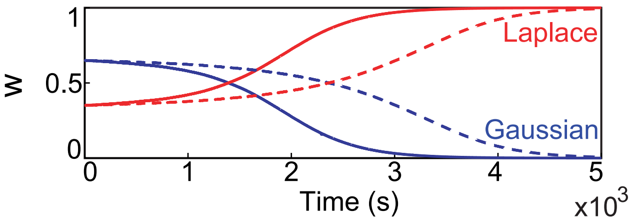

Figure 4. IP speeds learning. Evolution of weights for a Gaussian and a Laplacian input, with (solid line) and without IP (dotted line). In the second case, the neuron transfer function is fixed to the parameters obtained by IP at the time of convergence.

References

1. Triesch J (2007) Synergies between intrinsic and synaptic plasticity mechanisms. Neural Computation 19: 885-909.

2. Izhikevich E, Desai N (2003) Relating STDP to BCM. Neural Computation 15: 1511-1523.

3. Hyvärinen A, Karhunen J, Oja E (2001) Independent Component Analysis. Wiley Series on Adaptive and Learning Systems for Signal Processing, Communication and Control.

4. Oja E (1997) The nonlinear PCA learning rule in independent component analysis. Neurocomputing 17: 25-46.
