# Supplementary material for: Independent Component Analysis in Spiking Neurons
Source: PLoS Comput Biol. 2010 Apr 22;6(4):e1000757. doi: 10.1371/journal.pcbi.1000757 (PMC2858697; doi:10.1371/journal.pcbi.1000757)
Supplement: Text S4 — A link to BCM (0.15 MB PDF) [file pcbi.1000757.s004.pdf]

S4. A link to BCM

From an information-theoretic perspective, it is interesting to relate our approach to previous work on maximizing information transmission between neuronal input and output by optimizing synaptic learning [1]. This optimization procedure results in a spike-based rule that implements a generalized version of the classic BCM rule. A link between BCM and IP-based learning has been noted previously [2], when it has been argued that the slow sliding threshold of BCM could play a homeostatic role similar to that of IP. Here we address this question again, from the perspective of learning.

For this comparison, we use the minimal triplets STDP model [3]. When combined with an additional sliding threshold, this model was previously shown to exhibit input selectivity [3]. However, to our knowledge, the case when several independent sources are present in the input at the same time was not investigated before with spiking neurons.

The triplet model we consider here is a generalization of STDP learning, which takes into account the effects of spike triplets [3]. Under certain assumptions, it is equivalent to the model in [1], i.e. BCM-like, but computationally simpler. Specifically, the model computes one presynaptic (r_1) and two postsynaptic (o_1 and o_2) activity traces, with different time scales:

$$\begin{aligned}\frac{dr_1}{dt} &= -\frac{r_1}{\tau_+} + \delta(t - t^f_{\mathrm{pre}})\\ \frac{do_1}{dt} &= -\frac{o_1}{\tau_-} + \delta(t - t^f_{\mathrm{post}})\\ \frac{do_2}{dt} &= -\frac{o_2}{\tau_{\mathrm{y}}} + \delta(t - t^f_{\mathrm{post}})\end{aligned}$$

where δ is the Dirac function and $t^f_{\mathrm{pre/post}}$ is the time of firing of the pre- and post-synaptic neuron, respectively. The timescales of integration τ_+ and τ_- are similar to those in classical STDP ($\tau_+ = 16.8$ ms, $\tau_- = 33.7$ ms), while τ_{y} is slower ($\tau_{\mathrm{y}} = 114$ ms).

The change in weight can be computed as:

$$\frac{dw}{dt} = A_3^+ \cdot r_1 \cdot o_2 \cdot y(t) - A_2^- \cdot o_1 \cdot x(t) \cdot \frac{\Theta}{\Theta_{\mathrm{goal}}},$$

with the parameters $A_3^+ = 6.5 \times 10^{-4}$, $A_2^- = 7.1 \times 10^{-4}$ and $\Theta_{\mathrm{goal}} = 25$ is a scaling parameter which implicitly defines the goal mean firing rate of the neuron.

Here, we study the relation of IP-based and BCM learning by comparing two variants of the basic triplet rule, extended by a sliding threshold Θ that depends quadratically on the firing history or by intrinsic plasticity. For implementing sliding threshold BCM, the original triplet model model is enhanced with a slowly varying threshold Θ, which estimates a low-pass filtered version of the square of the postsynaptic firing rate, similar to the classical BCM sliding threshold [3]. Namely:

$$\frac{d\Theta}{dt} = -\frac{\Theta}{\tau_{\mathrm{th}}} + o_2^2,$$

with $\tau_{\mathrm{th}} = 100$ s.

When trying to compare IP-guided learning and sliding-threshold BCM one should note there is a fundamental difference between the two. While the sliding threshold of BCM has a direct effect on learning (by changing the threshold between potentiation and depression), IP affects plasticity only indirectly, through a change in neuronal output. As an illustration, consider the classic BCM weight change curve in Fig. 1A (blue curve), obtained with the minimal triplets STDP model described above. Unlike the standard representation, the horizontal axis represents the total input to the neuron u and not the postsynaptic firing rate. In the classic sliding threshold BCM framework, the threshold between

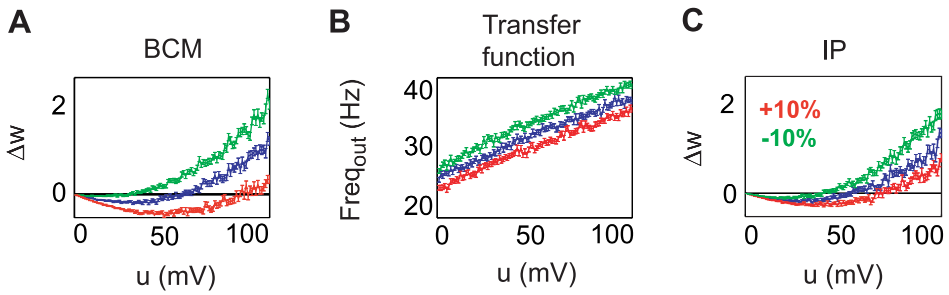

Figure 1. Comparing sliding threshold BCM with IP (A) Weight changes for the minimal triplets model. Depending on the neuronal history (by $f = \pm 10\%$), the threshold between potentiation and depression varies. (B) For the same variations in input (as before, the input is assumed to be Gaussian, with mean varying such that $x_1 = f \cdot x_0$) IP changes the neuronal transfer function to preserve the same neuronal output distribution ($u_\alpha^1 = f \cdot u_\alpha^0$ and $u_0^1 = f \cdot u_0^0 - (f-1) \cdot u_r$, see Methods). (C) Corresponding to the shift of the transfer function, the weight changes for the same input vary. All estimates were averaged over 100 trials, each lasting 1 second.

potentiation an depression shifts depending on the history of the neuron. However, the output of the neuron to the current sample remains the same. This threshold can be computed analytically for a linear neuron, assuming a change in the total input distribution by a factor f, as $\Theta' = f^2 \cdot \Theta$.

For the same alteration of the input distribution, IP causes a change in the transfer function (Fig. 1B), which also results in a shift of the synaptic learning curve along the horizontal axis (Fig. 1C), while the actual STDP threshold remains the same (the change in parameters by IP was computed analytically as $u'_\alpha = f \cdot u_\alpha$ and $u'_0 = f \cdot u_0$, with the same triplet STDP rule as before). The amplitude of this shift may depend on the IP parameters, but essentially it achieves the same type of alteration as sliding threshold BCM.

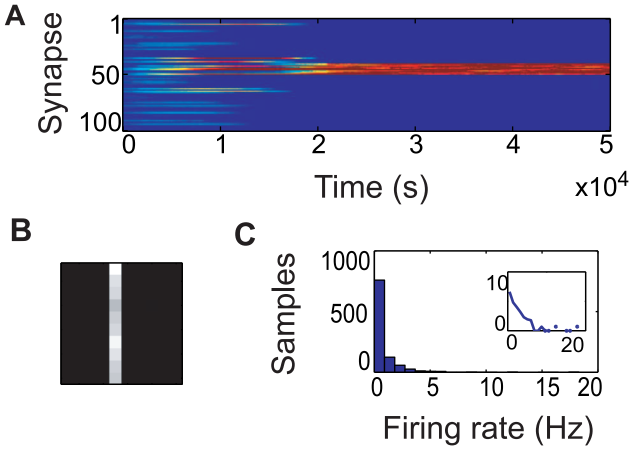

Figure 2. Learning an IC with sliding threshold BCM. (A) Evolution of input weights for the bars problem. (B) Final receptive field learned. (C) Distribution of firing rates of the neuron; inset with the same measure in logarithmic scale.

Given the similarity between our rule and BCM, and previous work showing the triplets STDP exhibit input selectivity [1,3], we hypothesized that a BCM rule with slowly-adapting threshold could also solve the bars problem. Indeed, as seen in Fig. 2, spiking neurons by BCM-like spike-based synaptic learning can extract an IC. Moreover, as expected, after learning neuron responses become sparse and the output distributions highly kurtotic (see Fig. 2C).

Several constraints are necessary for the development of a stable receptive field with the sliding-threshold BCM rule. Firstly, as in the case of IP, synaptic scaling is required for stabilizing the receptive field. This is somewhat surprising, as BCM theory would predict that no constraint on the weight vector should be needed. Secondly, the parameter Θ_{goal} must be adjusted to the input characteristics for a bar to be a stable solution. The obvious advantage of IP is that it is robust to parameters changes and does not require tight tuning of any parameter. Moreover, directly optimizing the neuron excitability allows for various synaptic learning implementations, which may make a combined approach more suitable given different biological constraints.

References

1. Toyoizumi T, Pfister J, Aihara K, Gerstner W (2005) Generalized Bienenstock-Cooper-Munro rule for spiking neurons that maximizes information transmission. PNAS 102: 5239-5244.

2. Triesch J (2007) Synergies between intrinsic and synaptic plasticity mechanisms. Neural Computation 19: 885-909.

3. Pfister J, Gerstner W (2006) Triplets of spikes in a model of spike timing-dependent plasticity. Journal of Neuroscience 26: 9673-9682.
